# Characterization of C3larvinA, a novel RhoA-targeting ADP-ribosyltransferase toxin produced by the honey bee pathogen, *Paenibacillus larvae*

**DOI:** 10.1042/BSR20193405

**Published:** 2020-01-10

**Authors:** Madison Turner, Olivier Tremblay, Kayla A. Heney, Miguel R. Lugo, Julia Ebeling, Elke Genersch, A. Rod Merrill

**Affiliations:** 1 Department of Molecular and Cellular Biology, University of Guelph, Guelph, Ontario N1G 2W1, Canada; 2 Institute for Bee Research, Department of Molecular Microbiology and Bee Diseases, Hohen Neuendorf 16540, Germany; 3 Freie Universität Berlin, Fachbereich Veterinärmedizin, Institut für Mikrobiologie und Tierseuchen, Berlin 14163, Germany

**Keywords:** ADP-ribosylation, bacterial toxins, honey bee diseases, microbial pathogenesis, virulence factors

## Abstract

C3larvinA is a putative virulence factor produced by *Paenibacillus larvae* enterobacterial-repetitive-intergenic-consensus (ERIC) III/IV (strain 11-8051). Biochemical, functional and structural analyses of C3larvinA revealed that it belongs to the C3-like mono-ADP-ribosylating toxin subgroup. Mammalian RhoA was the target substrate for its transferase activity suggesting that it may be the biological target of C3larvinA. The kinetic parameters of the NAD^+^ substrate for the transferase (*K*_M_ = 75 ± 10 µM) and glycohydrolase (GH) (*K*_M_ = 107 ± 20 µM) reactions were typical for a C3-like bacterial toxin, including the Plx2A virulence factor from *Paenibacillus larvae* ERIC I. Upon cytoplasmic expression in yeast, C3larvinA caused a growth-defective phenotype indicating that it is an active C3-like toxin and is cytotoxic to eukaryotic cells. The catalytic variant of the Q187-X-E189 motif in C3larvinA showed no cytotoxicity toward yeast confirming that the cytotoxicity of this factor depends on its enzymatic activity. A homology consensus model of C3larvinA with NAD^+^ substrate was built on the structure of Plx2A, provided additional confirmation that C3larvinA is a member of the C3-like mono-ADP-ribosylating toxin subgroup. A homology model of C3larvinA with NADH and RhoA was built on the structure of the C3cer-NADH-RhoA complex which provided further evidence that C3larvinA is a C3-like toxin that shares an identical catalytic mechanism with C3cer from *Bacillus cereus*. C3larvinA induced actin cytoskeleton reorganization in murine macrophages, whereas in insect cells, vacuolization and bi-nucleated cells were observed. These cellular effects are consistent with C3larvinA disrupting RhoA function by covalent modification that is shared among C3-like bacterial toxins.

## Introduction

Many insect species are indispensable for the pollination of wild and cultivated plants and therefore essential for both the survival of natural ecosystems and for a sufficiently diverse human diet [1,2]. Among these insect species, honey bees play a prominent role because managed colonies are invaluable agents for targeted pollination in agriculture [3]. In fact, commercial pollination of fruit and crops is a pillar to agricultural prosperity extolling the economic value of honey bee colonies in many regions of the world [4,5]. Given the importance of honey bees in human life and prosperity, it is not surprising that they have received much scientific attention. Honey bee pathogens, however, are still a niche existence in science. Exceptions to this rule are those pathogens that cause considerable colony losses worldwide [6–8], like the ectoparasitic mite *Varroa destructor* [9], the bee pathogenic viruses vectored by the mite [10,11], and *Paenibacillus larvae* (*P. larvae*), the causative agent of American Foulbrood (AFB) [12,13].

AFB is a lethal, highly contagious, globally distributed bacterial disease of the honey-bee brood and a notifiable epizootic in most countries [14]. AFB is a common bee disease–most authorities consider a strict destructive policy toward clinically diseased colonies as the only workable measure in disease management. Importantly, AFB is responsible for substantial annual economic losses in apiculture [15]. Over the past decade, knowledge of AFB pathogenesis at the larval level, including the mechanism of *P. larvae* invasion and associated disease symptoms in the honey-bee brood, has improved tremendously [16]. The *P. larvae* species comprise four different so-called enterobacterial-repetitive-intergenic-consensus (ERIC)-genotypes [13] which have been named according to the ERIC primers used for differentiation via repetitive element PCR (repPCR) [17]. The genotypes differ in their overall genetic makeup [18], but also in their phenotypes [13,19]. Phenotypic variation includes differences in virulence and pathogenic strategies [20,21] and are best analyzed for the *P. larvae* genotypes, ERIC I and ERIC II, which are the strains isolated from contemporary AFB outbreaks worldwide. For these two genotypes, several virulence factors have been both biochemically and functionally characterized in the recent past. General virulence factors common to both genotypes are the chitin-degrading enzyme *Pl*CBP49 [22], which is responsible for the destruction of the peritrophic matrix as a key step in killing the larvae [23], and several secondary metabolites [24] acting against microbial competitors in the larval gut [25,26] or serving as iron scavengers to meet iron limitation in the host [27]. Genotype-specific virulence factors for *P. larvae* ERIC II are specific secondary metabolites with antibacterial [28] and antifungal activity [29,30] or facilitating swarming behavior [31]. The most prominent ERIC II-specific virulence factor is the surface layer (S-layer) protein, SplA, which mediates *P. larvae* adhesion to the midgut epithelium, a step that might initiate breaching the epithelial cell layer leading to larval death [32,33]. No functional toxin gene loci were annotated in the genome of *P. larvae* ERIC II [34]. In contrast, comparative whole genome analysis [34] confirmed early results suggesting that *P. larvae* ERIC I genomes harbor functional toxin genes [18]. Among the toxin loci found in the genome of *P. larvae* ERIC I, only a few were considered functional [34]. Those included the loci encoding two toxins, Plx1 and Plx2, which had previously been demonstrated to act as ERIC I-specific virulence factors [35]. Based on their overall structure, both toxins, Plx1 and Plx2, were classified as mono-ADP-ribosylating toxins [35].

In the interaction between bacterial pathogens and their hosts, bacterial exotoxins often play an important role. It is well established that secretion of toxin proteins by viable pathogenic bacteria contributes to tissue damage and disease symptoms as well as facilitates replication and transmission of the bacteria to new hosts. Exotoxins can be broadly divided into three types–toxins that signal at host cell membranes (type I), toxins that act on and destroy host cell membranes (type II), and toxins that overcome the host cell membrane, enter the host cells, and directly alter host cell function by modifying intracellular target molecules (type III). One of the most common modifications is ADP-ribosylation of cellular targets by type III toxins exhibiting mono-ADP-ribosyltransferase (mART) activity. This enzymatic activity, contained in the A-subunit of the protein, is the only unique feature among ADP-ribosylating toxins; otherwise, they are unrelated in their structure and form three classes of toxins: A/B toxins, binary toxins and A-domain-only toxins. In A/B toxins, a single protein contains both the enzymatically active A-domain and the B-domain, which binds the appropriate cell-surface receptor and mediates the translocation of the A-domain into the host cell cytoplasm. In contrast, binary toxins are composed of two separate protein subunits, the enzymatically active A-subunit and the translocating B-subunit. The third class, the A-domain-only toxins, are single domain exoenzymes consisting only of the A-domain and lacking an associated B-domain or B-subunit. In most cases, their mechanism of cell entry is not known.

In the literature, C3-like mARTs are described as single-domain exoenzymes produced exclusively by four species of Gram-positive pathogens, *Clostridium botulinum, C. limosum, Bacillus cereus*, and various strains of *Staphylococcus aureus* [36]. Recently, a C3-like toxin was found in a fifth bacterial species in the Gram-positive bacterium, *P. larvae*, a well-known honey-bee pathogen. This C3-like toxin, Plx2A, is encoded by a toxin locus present only in representatives of the *P. larvae* genotype ERIC I [18,34,35]. *In silico* analysis of the Plx2A locus suggested that this toxin could be an exception to the rule that all C3-like toxins are A-domain-only toxins because the Plx2 locus comprises two genes, one coding for Plx2A and an adjacent, upstream located gene coding for a putative B-subunit, Plx2B [35]. Experimental evidence based on laboratory infection of honey bee larvae with wild-type (WT) *P. larvae* and corresponding gene inactivation mutants for *plx*2A and *plx*2B, demonstrated that both the A- and the B-subunits act as virulence factors during pathogenesis [35]. Biochemical characterization of Plx2A for cytotoxicity, enzymatic activity, RhoA target recognition, and cell invasion, as well as the Plx2A crystal structure, unequivocally confirmed that it is a C3-like exoenzyme [37]. Incubating insect cells with purified Plx2A revealed that although the toxin targets RhoA, it does not interfere with the actin cytoskeleton as observed for the other C3-like toxins in mammalian cells, but inhibits cytokinesis resulting in enlarged, binucleated cells [37]. The function of the putative B-subunit for cell entry and toxin activity remains elusive so far, particularly because it is not essential for Plx2A cytotoxicity in the insect cell culture assays.

Recently, yet another C3-like exoenzyme of *P. larvae*, (C3larvin) herein called C3larvin_trunc_, was discovered via genome mining, identified as a mART toxin that targets RhoA, and suggested to be a virulence factor for *P. larvae* ERIC I and ERIC II [38]. However, C3larvin_trunc_ was shown to lack N-terminal sequences responsible for cell-entry activity, and indeed, the toxin was unable to invade mouse macrophages [38]. Consistent with this observation, *P. larvae* ERIC I and ERIC II gene inactivation mutants lacking C3larvin_trunc_ expression did not cause larval mortality compared with WT strains when used for experimental infection [39]. These data suggested that despite its enzymatic activity in biochemical assays [38], C3larvin_trunc_ does not influence the virulence of *P. larvae* [39]. Further *in silico* analyses then revealed that in *P. larvae* ERIC I and ERIC II, the *C3larvin_trunc_* gene is part of a binary AB toxin locus which had been annotated as non-functional due to several disruptions of the open-reading frames coding for the A- and B-subunits [34]. In this context, a full-length C3larvinAB locus comprising non-interrupted genes for the A- (C3larvinA) and the B-subunits (C3larvinB) was found in a singular *P. larvae* ERIC III/IV strain. Remarkably, C3larvinA contains the N-terminal sequences [39] not present in the originally described, inactive C3larvin_trunc_ [38].

Herein, we now report the biochemical and functional characterization of C3larvinA as a full-length, C3-like toxin virulence factor (a full-length version of C3larvin_trunc_) [38] produced by *P. larvae* ERIC III/IV strains. We show that C3larvinA has 55% sequence identity with Plx2A, the other functional C3-like toxin of *P. larvae* produced by ERIC I strains. C3larvinA binds and hydrolyzes NAD^+^ as substrate, has glycohydrolase (GH) activity, and enzymatically modifies RhoA with ADP-ribose. Hence, it is a classical mART enzyme and contains the hallmark catalytic motifs and residues conserved among C3-like toxins. Recombinantly expressed C3larvinA was used to characterize its functional and biochemical properties. Purified C3larvinA enters host cells and interferes with actin remodeling in mouse macrophages and cytokinesis in insect cells. Therefore, C3larvinA not only resembles Plx2A in that it has an associated B-subunit, but also shows the same phenotype in mammalian and insect cells as previously described for Plx2A [37]. Additionally, C3larvinA represents a full-length version of the previously characterized C3larvin_trunc_ and can enter host cells. C3larvin_trunc_ was unable to enter macrophages because it is an inactive toxin at the cell entry level due to an N-terminal truncation (missing most of the α-1 helix). Furthermore, C3larvinA and Plx2A are functionally and structurally similar virulence factors that originate from different *P. larvae* strains; C3larvinA is produced by *P. larvae* ERIC III/IV strains, while Plx2A is produced by ERIC I strains.

## Experimental procedures

### Transformation, expression, and purification

The C3*larvinA* gene was cloned into a pET-28a^+^ vector with an N-terminal hexa-histidine tag. This plasmid was used to transform chemically competent *Escherichia coli* BL21 λDE3 cells using the heat-shock method. The cells were then grown in 4 l of 2× YT media containing 30 µg/ml kanamycin at 37°C. When the culture reached an OD_600_ of 0.6, expression of C3larvinA was induced with 1 mM IPTG. The culture was grown for an additional 3 h at 37°C before being harvested by centrifugation at 3000×***g*** for 15 min at 4°C. The cell pellet was resuspended in buffer containing 500 mM NaCl and 50 mM Tris/HCl at pH 7.5. The cells were then lysed using an Emulsiflex-C3 high pressure homogenizer (Avestin Inc., Ottawa, Canada) in the presence of 120 μM PMSF, 50 μg/ml CHAPS, 1 mM EDTA, and 100 μg/ml DNase. Next, the homogenate was centrifuged at 23700×***g*** for 55 min at 4°C to remove insoluble cell debris. The supernatant was then incubated with 10 mM MgCl_2_ with mixing for 30 min at 4°C. This solution was passed over a Ni^2+^-charged chelating FastFlow™ Sepharose column, which was then washed twice, first with lysis buffer containing 25 mM imidazole, and second, with lysis buffer containing 40 mM imidazole. A final wash with lysis buffer containing 250 mM imidazole was used to elute C3larvinA. Fractions were analyzed with SDS/PAGE, and fractions showing bands at the correct molecular weight for C3larvinA were dialyzed overnight in lysis buffer and further purified using a HiLoad 16/60 Superdex-200 column (GE Healthcare) in size-exclusion chromatography (SEC). Fractions showing pure protein after being analyzed via SDS/PAGE were pooled and concentrated.

### Site-directed mutants of C3larvinA

Point mutations were introduced into the *C3larvinA* gene using the QuikChange® Mutagenesis method (Stratagene, La Jolla, CA, U.S.A.) according to the manufacturer’s instructions. The Gln and the Glu of the Q^187^-X-E^189^ motif (catalytic center and major site) were exchanged individually in single variants (Q187A and E189A), or together in a double variant at residues Gln and Glu (A-X-A) by substitution with Ala. The STS motif (residues Ser^149^–Thr^150^–Ser^151^) (NAD^+^ substrate binding site) was substituted with three Ala residues (A-A-A). The catalytic Arg residue was also substituted with Ala (R104A). The C3larvinA variants (Table 1) were expressed and purified as described for the WT C3larvinA.

**Table 1:** Melting temperatures of C3larvinA and catalytic variants

Protein	Melting temperature (°C)^1^
C3LarvinA	63 ± 0.3
C3larvinA AAA	62 ± 0.4
C3larvinA R105A	60 ± 0.3
C3larvinA AXA	62 ± 0.2

1 The melting temperatures (*T*_m_) of the C3larvinA WT and variants are described in the ‘*Experimental procedures*’ section. The *T*_m_ values represent the mean ± standard deviation (SD) of at least three measurements.

### Circular dichroism spectroscopy

C3larvinA and catalytic variants were dialyzed into buffer containing 250 mM NaF and 10 mM Tris/HCl, pH 7.5. A JASCO J‐815 circular dichroism (CD) spectropolarimeter was used to acquire the CD spectra of C3larvinA WT and variant proteins (0.16 mg/ml) at 25°C in a 1-mm pathlength cuvette by scanning from 250 to 190 nm for a total of nine scans from which an average spectrum was calculated.

### Expression and purification of RhoA ΔCAAX-GST

RhoA does not express well in soluble form unless as a GST-fusion protein. Therefore, RhoA–GST fusion proteins are typically used for toxin kinetic analyses when RhoA is the putative cellular target [36,40]. Constitutively active human GST-RhoA (ΔCAAX) was recombinantly expressed in *E. coli* TG1 from a plasmid obtained as a gift from Dr. Joseph Barbieri (Medical College of Wisconsin) and purified essentially as described previously [38]. Briefly, RhoA ΔCAAX (C = cysteine, A = aliphatic amino acid, X = any amino acid) - GST expression was started by inoculating a 2× YT culture including ampicillin (100 µg/ml) with transformed *E. coli* TG1. Bacteria were grown until an OD_600_ of 1 was reached at 37°C. Expression was induced with 1 mM IPTG at 27°C overnight. The cultures were centrifuged at 3000×***g*** at 4°C for 12 min. Afterward, the pellets were dissolved in lysis buffer (10 mM HEPES, pH 7.5, 150 mM NaCl, 2.5 mM MgCl_2_, 1 mM DTT) including freshly added DNase. The pellet was lysed by sonication or high-pressure homogenization, and cell debris was removed by centrifugation. The supernatant was passed three times over a column containing glutathione resin (GenScript, Piscataway, NJ, U.S.A.) calibrated with lysis buffer. The column was washed five times with lysis buffer and the recombinant protein eluted with lysis buffer containing 20 mM reduced glutathione. To remove the glutathione, the purified protein was dialyzed into lysis buffer at 4°C overnight.

### Homology model of C3larvinA

**Figure 1:**
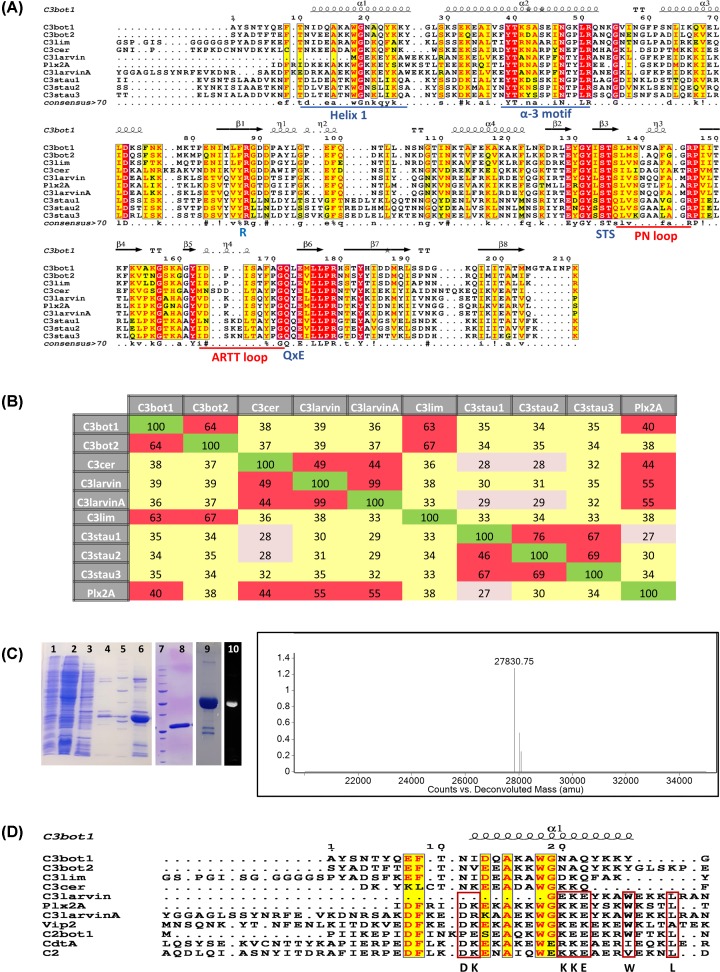
Multiple-sequence alignment of the C3 toxin subgroup (**A**) Multiple sequence alignment of C3 toxins and C3larvinA using the T-Coffee web server to align the sequences and ESPript to generate the figure [70]. Key catalytic regions are highlighted. Identical residues are highlighted in red, and similar residues are printed in red text and highlighted in yellow. The α-3 motif, helix 1, and the ARTT and PN-loops are indicated by underlined sequences. The three catalytic motifs in C3 toxins are indicated below the corresponding sequences. (**B**) Identity matrix showing the amino acid identity between the 100 core catalytic residues of the known C3 toxins and C3larvinA. Red, yellow and gray shading indicates high, medium and low sequence identities among C3 toxin pairs. The identity matrix was generated using ClustalX2 [71] and colored using Microsoft Excel. (**C**) Left side: purification and identification of C3larvinA from *E. coli* lysate. SDS/PAGE gels showing the purification and identification of C3larvinA. Lane 1, induced *E. coli* cell pellet; lane 2, IMAC sample flow-through; lane 3, column wash #1; lane 4, column wash #2; lane 5, Bio-Rad MW standards, 10-, 15-, 20-, 25-, 37-, 50-, 75-, 100-, 150-, 250-kDa; lane 6, partially purified C3larvinA as IMAC elution fraction; lane 7, Bio-Rad MW standards, Bio-Rad MW standards, 10-, 15-, 20-, 25-, 37-, 50-, 75-, 100-, 150-, 250-kDa; lane 8, purified C3larvinA after SEC; lane 9, Coomassie-stained RhoA reacted with C3larvinA and fluorescein-NAD^+^; lane 10, fluorescence image of lane 9 showing the fluorescence of the RhoA band. Right side: Q-TOF mass analysis of purified C3larvinA protein showing a single peak at 27830.75 Da, corresponding to the expected mass of recombinant C3larvinA (27830.66 Da). (**D**) Multiple sequence alignment of selected C2 and C3 toxins, and C3larvinA using the T-Coffee web server to align the sequences and ESPript to generate the figure [70]. Identical (or nearly so) residues between both C2 and C3 toxins are printed in red text and highlighted in yellow; identical (or nearly so) residues shared among the C2 toxins with *P. larvae* toxins, C3larvin_trunc_, Plx2A and C3larvinA, are bound by red rectangles. Abbreviations: IMAC, immobilized-metal-affinity chromatography.

**Figure 2:**
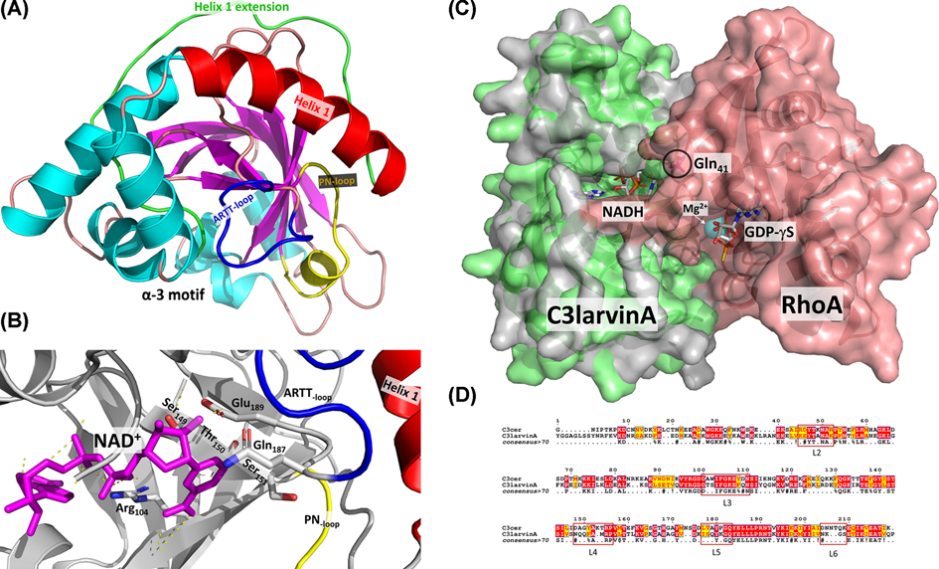
C3larvinA structures (**A**) C3larvinA homology model structure based on Plx2A from *P. larvae* (PDB: 5URP) is shown as a ribbon diagram. The NAD^+^ substrate was modeled within the active site of C3larvinA as described in the ‘*Experimental procedures*’ section. Secondary structural elements such as α-helices are colored in cyan and β strands are shown in magenta. The α-3 motif, helix 1 (red), the helix 1 N-terminal extension (green), and the ARTT- (dark blue) and PN-loops (yellow) are also labeled. (**B**) C3larvinA active-site catalytic elements are shown. Catalytic residues Arg^104^, Gln^187^ and Glu^189^ are shown in stick format with standard element colors. The STS motif (S_149_-T_150_-S_151_) is also shown in stick format, along with the PN-loop (yellow), ARTT-loop (dark blue) and helix 1 (red). The NAD^+^ substrate is bound in the active site and is shown in magenta with important H-bonds indicated by yellow dashed lines. (**C**) C3larvinA-NADH-RhoA homology model built on the C3cer-NADH-RhoA crystal structure (PDB:4XGS) is shown in surface rendering. C3larvinA is shown in light green and RhoA in salmon color. The NADH inhibitor is bound in the active site and is shown in stick format colored with standard element colors, Gln41 in RhoA is circled and is shown in stick format colored magenta; Mg^2+^ is shown as a cyan sphere and GDP-γS is shown in stick format with standard element colors. (**D**) Sequence alignment of C3cer and C3larvinA C3-like toxins using the T-Coffee web server to align the sequences and ESPript to generate the figure [70]. Identical residues between both C3-like toxins are printed in red text and highlighted in yellow; the conserved loop regions that form the critical interactions with the RhoA substrate based on the C3cer-NADH-RhoA crystal complex (PDB:4XGS) are bounded by red rectangles [40].

A homology model of C3larvinA was built based on the 1.65 Å Plx2A crystal structure (PDB: 5URP, apo/substrate-free; 55% sequence identity, Figures 1B and 2A) using Phyre2 (**P**rotein **H**omology/**A**nalog/**Y R**ecognition **E**ngine) [41] and was reported with 100% confidence. The Phyre2 method uses template detection by HHpred 1.51 [42], secondary structure prediction with Psi-pred 2.5 [43], disorder prediction using Disopred 2.4, and multi-template modeling and *ab initio* with Poing 1.0 [44]. The Plx2A structure was chosen as the template rather than C3larvin_trunc_ because the latter lacks a full helix 1 sequence whereas the former has a functional helix 1 [37]. The homology model includes the entire polypeptide chain of C3larvinA, but not the recombinant His-tagged, N-terminal region) and shares similar topology with the other C3-like toxins (Figure 2B). It is identical (99% sequence identity) with C3larvin_trunc_ from ERIC I and II *P. larvae* strains except for an extended N-terminal region which includes a full-length helix 1 (Figures 1A and 2B). In order to correctly model the helix 1 region within C3larvinA, Plx2A was chosen to provide a suitable template to model this helix (Arg^27^–Trp^41^ in C3larvinA) and to position the nearly conserved Phe^23^ and semi-conserved Glu^25^ residues located further upstream of helix 1 and which are found in the C3-subgroup (Figure 1A) except the C3larvin_trunc_ sequence. The C3bot1–NAD^+^ complex (PDB: 1GFZ.A) was used as the basis to model in the NAD^+^ substrate into the active site of C3larvinA [40].

### Force-field settings and structure preparation

Protein preparation and molecular mechanics (MM) calculations were performed using the computational suite Molecular Operative Environment (MOE) release 2018.10 (Chemical Computing Group Inc, Montreal, CA). The force field employed was the MOE Amber12:EHT, with AMBER12 parameters set (ff12) for protein, and parameters calculated from the Extended Hückey Theory for the NAD^+^ molecule. For the implicit solvent model, the Generalized Born-Volume Integral (GB/VI) formalism was employed, with dielectrics *ε_pro_* = 1 for the interior of the protein. The MOE Protonate3D module was used to assign the ionization states and tautomers of side-chains at T = 300 K, pH = 7.4 and 0.1 M of ionic strength, along with the GB/VI solvation model and MMFF94 partial charges. The *molecular surfaces* are solvent-excluded surfaces obtained by rolling a probe sphere of 1.4 Å diameter (water radius) and colored by several schemes. The *van der Waals-interaction surfaces* correspond to zero-potential contours of the van der Waals potential, *E_vdw_* = 1, between the specific set of atoms and a water O-atom as mobile probe, using a standard 12-6 Lennard-Jones definition.

### Modeling the C3larvinA–NAD^+^ complex

The NAD^+^ molecule was taken from the C3bot1–NAD^+^ complex (PDB:2C8C) and docked (translated) into the protonated apo C3larvin homology model built on the X-ray structure of Plx2A (PDB: 5URP) after optimal superposition of both proteins by their C_α_-atoms of pocket residues. Backbone atoms of this initial C3larvinA–NAD^+^ complex were fixed, and the system was energy minimized (root-mean-square (RMS) gradient ≤ 0.001 kcal/mol/Å^2^) in an implicit solvent model (*ε_sol_* = 80). Then, the energy function was updated to vacuum (*ε_sol_* = 80), and the MOE Solvate module was used to (*i*) locate the center of mass of the toxin at the center of a periodic box of 69.17 × 60.16 × 47.75 Å^3^ (edge lengths), (*ii*) solvate the complex with 6485 TIP3P water molecules at a density of 1.023 g/cm^3^, and (*iii*) neutralize the system by incorporating nine Cl^−^ ions at optimal locations. The system was energy minimized in a stepwise fashion (each to a RMS gradient ≤ 0.01 kcal/mol/Å^2^) as follows: first, the complex was fixed and the solvent (water and ions) was relaxed, then backbone atoms were fixed and side-chains, NAD^+^, and solvent were energy minimized, and finally the full system was minimized. With this molecular system, a molecular dynamics (MD) simulation was performed by the Scalable MD (NAMD) simulator release 2.9 [45], under periodic boundary conditions by wrapping protein and solvent, with an integration time of 1 fs and recording each 5 ps under the following sequential steps: (*i*) 1000 ps heating from 0 to 295 K; (*ii*) 4000 ps equilibration at 295 K; and finally (*iii*) an NPT ensemble at 295 K and 1 atm of production phase for 100 ns. Then, 20000 frames of the MD trajectory were stripped off solvent molecules and calculated the potential energy of the C3larvinA–NAD^+^ decoys under an implicit solvent with *ε_sol_* = 80. All decoys with potential energy lower than the average value were selected to an energy minimization (RMS gradient ≤ 0.01 kcal/mol/Å^2^). The decoy with the lowest potential energy after this geometry optimization step was saved and reported as the ‘C3larvinA–NAD^+^ complex’ model.

### Homology model of C3larvinA–NADH–RhoA complex structure

A homology model of C3larvinA-NADH-RhoA complex was built based on the 1.8 Å C3cer exoenzyme-NADH-RhoA crystal structure (PDB:4XSG; 48% sequence identity) [40] (Figure 2C) using Phyre2 [41] and was reported with 100% confidence. The Phyre2 method uses template detection by HHpred 1.51 [42], secondary structure prediction with Psi-pred 2.5 [43], disorder prediction using Disopred 2.4, and multi-template modeling and *ab initio* with Poing 1.0 [44].

### Differential-scanning fluorimetry

The thermal stability of C3larvinA WT and variants was measured in triplicate measurements by melting-curve analysis in a StepOnePlus Real-time PCR system (Applied Biosystems, Foster City, CA, U.S.A.) using Protein Thermal Shift dye, Sypro Orange® according to the manufacturer’s instructions (Applied Biosystems) adopted from a previous method [46,47]. Melting curve analysis of the purified proteins established that C3larvinA and all active-site variants had a single, distinct melting point and similar melting temperature values (*T*_M_) (Table 1) indicating that all purified proteins were stable and properly folded.

### GH activity

The GH activity of C3larvinA against etheno-NAD^+^ (ε-NAD^+^) was measured on a Cary Eclipse fluorescence spectrometer (Agilent Technologies, Mississauga, Canada) with 305 nm excitation and 405 nm emission wavelengths, and bandpasses of 5 nm. C3larvinA at 20 μM and ε-NAD^+^ concentrations ranging from 0 to 500 μM in reaction buffer (50 mM NaCl and 20 mM Tris, pH 7.9) were mixed in a total volume of 75 μl. The reaction was monitored for 5 min, and the resulting slope was converted from relative fluorescence units into µM concentrations using a standard etheno-AMP (ε-AMP) curve. All Michaelis–Menten kinetics values were calculated from initial rate data using GraphPad ver 8 Software (La Jolla, CA, U.S.A.).

### NAD^+^ substrate binding

The affinity between C3larvinA and β-NAD^+^ was assessed in a tryptophan fluorescence-quenching assay using a Cary Eclipse fluorescence spectrometer with 295 nm excitation and 340 nm emission, and 5 nm bandpasses. A solution of 1.25 μM C3larvinA protein in 600 μl buffer (20 mM Tris, pH 7.9, 50 mM NaCl) was titrated with β-NAD^+^ concentrations between 1 and 1000 μM. The average of all the standard curve slopes was considered for the conversion of fluorescence units/min of the initial sample slope into [ε-ADP-ribose] formed/min. The converted slopes were plotted against the ε-NAD^+^ concentration and fitted to the Michaelis–Menten model using GraphPad Prism Ver 8 software to calculate the kinetic parameters. The assay was repeated in triplicate with three technical replicates for each sample.

### Transferase activity

The ADP-ribosylation activity of C3larvinA against RhoA-GST was measured using an end-point assay. It was not possible to monitor the modification of RhoA-GST with ADP-ribose since this reaction product is unstable. The second product of the C3larvinA-catalyzed transferase reaction to RhoA-GST, nicotinamide, was measured using an Agilent high-performance liquid chromatography (HPLC) system. To measure the kinetic parameters in relation to RhoA-GST, β-NAD^+^ was held at 300 μM to ensure saturation while varying the concentration of RhoA-GST from 0 to 80 μM. Conversely, to measure parameters in relation to β-NAD^+^, RhoA-GST was held at 20 μM to ensure saturation while varying the concentration of NAD^+^ from 0 to 500 μM. The transferase reaction was conducted in buffer containing 5 mM MgCl_2_, 150 mM NaCl and 20 mM Tris/HCl, pH 7.4. The reaction was initiated with the addition of 1 μM C3larvinA and was stopped after 5 min with the addition of mobile-phase solution, including an internal standard (5% acetonitrile and 95% of 20 mM monobasic phosphate buffer pH 5.5, and 2.5 μg/ml para-4-nitrobenzoic acid; PABA). This produced a final ratio of 25% sample to 75% mobile phase (v/v). The solutions were passed through a Captiva filtration 96-well plate (Agilent Technologies) to remove the enzyme protein and then the sample was injected on to a Zorbax RX-C18, 5 μm, 4.6 mm × 12.5 mm reversed-phase column operating at 0.8 ml/min at 85 bar with 259 nm detection (Agilent Technologies, Mississauga, ON, Canada). An isocratic run of 10 min proved successful at separating the reaction components. A product standard curve was generated using various concentrations of nicotinamide (0–750 μM in mobile phase buffer) that were injected into the HPLC system. The area under the nicotinamide peak was determined using the peak analysis function in Origin 8.0 (Northampton, MA) and was standardized with the PABA internal standard. Background GH activity was corrected for each sample, and the calibrated area was converted into pmole of nicotinamide using a standard nicotinamide curve. Kinetic values were calculated using GraphPad version 8 software.

### Yeast cytotoxicity assay

Toxicity of C3larvinA WT and catalytic variants was tested on *Saccharomyces cerevisiae* BY4741 (*MATa, his3Δ1 leu2Δ0 met15Δ0 ura3Δ0*) using a yeast growth-deficiency assay as previously described [37,48]. Briefly, electrocompetent *S. cerevisiae* cells were prepared according to a modified protocol [49]. Cells were co-transformed with linearized pRS415-*CUP1* vector and a *larvinA* insert flanked by short sequences homologous to the vector. Transformant cells containing the pRS415-*CUP1-larvinA* vector formed through homologous recombination were incubated overnight in SD-LEU selective media and diluted to OD_595_ = 2 × 10^−4^. Expression of C3larvinA was induced by adding CuSO_4_ to a final concentration of either 0, 0.25, 0.5, or 0.75 mM. Cultures were dispensed into a 96-well plate such that each concentration of CuSO_4_ was repeated in 12 biological replicates, each consisting of 4 technical replicates. Sealed 96-well plates were incubated at 30°C for 48 h. Absorbance measurements were taken at 595 nm using a FLUOstar® Omega microplate reader (BMG LABTECH, Ortenberg, Germany). The catalytic domain of *Pseudomonas aeruginosa* ExoA was used as a positive control for the yeast growth-defective phenotype.

### Macrophage cytotoxicity assay

J774A.1 murine macrophage cells were grown in Dulbecco’s Modified Eagle’s Medium with 10% fetal bovine serum in the presence of penicillin–streptomycin antibiotic. Cells were grown in 25-cm^2^ breathable flasks with 5% CO_2_ and passaged at 80–90% confluence. Cells were lifted through scraping and diluted ten-fold into the above medium. Confluent J774A.1 cells were used to assess the effect of C3larvinA cellular processes. Cells were diluted to 2.5 × 10^5^ cells/ml in the presence of either 30 or 300 nM toxin concentrations. One hundred and fifty microliters of cell suspension was added in triplicate to a 96-well plate and allowed to incubate for 20 h. After this time period, cells were viewed under a microscope and photographed.

### Insect cell culture and actin staining

In the present study, the cell line Sf9 derived from *Spodoptera frugiperda* (Lepidoptera) was used to analyze the effect of C3larvinA on insect cells. Sf9 cells were cultured at 27°C in Insect-XPRESS w/L-Gln medium (Lonza, Verviers, Belgium) supplemented with 5% heat inactivated fetal bovine serum (Gibco, Thermo Fisher Scientific). Before the start of the assay, cells were grown to confluence. The cell number was determined in a Neubauer improved counting chamber. Cells were diluted to 0.5 × 10^5^ cells/ml in medium supplemented with penicillin (250 IU/ml)–streptomycin (250 µg/ml) (Carl Roth GmbH and Co. KG, Karlsruhe, Germany). The cells were premixed with sterile filtered, purified C3larvinA with a final concentration near 1.0 µM in reference to the C3-like toxin Plx2A as previously described [37]. The negative control contained sterile filtered NaCl-Tris-buffer with an equal volume to the purified C3larvinA. A volume of 2.0 ml of each mixture was added to wells of a sterile Cellstar six-well tissue culture plate (Greiner Bio-One GmbH, Kremsmünster, Austria) which were previously equipped with a sterile coverslip. The cells could adhere for 2 h on the bench and then incubated at 27°C for 2 days. For actin staining, the cells were fixed in Roti-Histofix 4% (Carl Roth GmbH and Co. KG, Karlsruhe, Germany) on the coverslip. The actin cytoskeleton was stained with Phalloidin Control, DyLight 488 conjugate (Invitrogen, Thermo Fisher Scientific, Darmstadt, Germany). To also stain the cell nuclei, the coverslips were mounted by using ProLong Gold Antifade Mountant with DAPI (Invitrogen, Thermo Fisher Scientific, Darmstadt, Germany). The cells were analyzed with a Nikon Ti-E Inverted Microscope (Düsseldorf, Germany) with NIS Elements AR 3.10 Software (Laboratory Imaging, Nikon, Düsseldorf, Germany) by using Differential Interference Contrast (DIC) for light microscopy and a FITC or DAPI filter for the appropriate fluorescence detection.

## Results

### *P. larvae* ERIC III/IV strain, 11-8051, encodes a C3-like mART toxin

The 248-residue (28.6 kDa) C3larvinA protein produced by *P. larvae* strain ERIC III/IV strain, 11-8051 [39] is a single-domain mART toxin that possesses a 26-residue leader sequence. The mature protein is 222 residues (25.7 kDa). It has a B-subunit partner that encodes a 562-residue, 62.2 kDa protein. C3larvinA has the hallmark catalytic Q-X-E signature of C3-like mART toxins (Figure 1A) [39]. The ten C3-like exotoxins known so far are shown in the sequence identity matrix (Figure 1B) and share a minimum of 27% sequence identity, and the most similar pair includes C3larvin_trunc_ and C3larvinA at 99% identity (Figure 1A,B). Unlike C3larvin_trunc_ from ERIC I and II *P. larvae* strains, C3larvinA possesses an intact Helix 1 and is the full-length and biologically active C3-like toxin [39] (Figure 1A,B). All C3-like enzymes are A-domain only toxins, except for Plx2 toxin, a major virulence factor exclusively expressed by *P. larvae* ERIC I strains [50]. Plx2A (A-domain only) shares 55% sequence identity with C3larvinA (Figure 1B) and was recently characterized for enzymatic activity, RhoA target recognition, cell invasion, and its crystal structure was also determined [37]. The C3-like proteins all share a conserved α-3 motif, catalytic Arg, S-T-S motif, PN-loop, ARTT loop and catalytic Q-X-E motif (Figure 1A). Additionally, the C3-like enzymes consist of a small mART domain (<25 kDa) and have both transferase and GH activity, although GH activity is considerably weaker than transferase activity [36,51].

Most C3-like toxins modify the small G-proteins, RhoA, RhoB, and RhoC, at the Asn^41^ residue [36,51]. RhoA ADP-ribosylation occurs at the highest rate, followed by that of RhoB and then RhoC [52]. Rac and Cdc42 are targets of several C3-like toxins, but show weaker ADP-ribose substrate activity than RhoA [36,53]. C3stau1, 2, and 3 are C3-like toxins which modify RhoE and Rnd3 as well as RhoA, B, and C; however, C3stau toxins show weaker transferase activity against all other protein substrates compared with RhoA [36,53].

### Expression of C3larvinA in *E. coli*

The C3larvinA (*LarA*) gene product was overexpressed in Rosetta *E. coli BL21(λDE3)* cells and the WT protein was purified at a yield of 1 mg/l culture using immobilized-metal-affinity chromatography (IMAC) followed by SEC. The purity level was assessed by SDS/PAGE (Figure 1C, lanes 6 and 8 for IMAC and SEC, respectively), and the recombinant 27.8 kDa protein (N-terminal His_6_ tag with TEV8 cleavage site) was positively identified by MALDI-TOF mass spectrometry (Figure 1C spectrum).

### C3larvinA structure

A homology model of C3larvinA with NAD^+^ substrate bound in the active site was built based on the 1.65 Å Plx2A crystal structure (PDB: 5URP, apo/substrate-free; 55% sequence identity, Figure 1B) (Figure 2A) using Phyre2 [41] The homology model includes the entire polypeptide chain of C3larvinA (but not the recombinant His-tagged, N-terminal region) and shares similar topology with the other C3-like toxins (Figure 2A,B). It is identical (99% sequence identity) with C3larvin_trunc_ from ERIC I and II *P. larvae* strains except for an extended N-terminal region which includes a full-length helix 1 (Figures 1A and 2A,B). It folds into a mixed α/β structure with a β-sandwich core and displays a characteristic mART fold, containing two perpendicular β-sheets next to the P–N loop, responsible for binding the NAD^+^ substrate (Figure 2A,B). Superposition of C3larvinA with the apo conformations of C3bot1 (PDB: 1G24), C3stau2 (PDB: 1OJQ), C3lim (PDB: 3BW8), Plx2A (PDB: 5URP) and C3larvin_trunc_ (PDB: 4TR5) revealed low root mean square deviations (r.m.s.d) of 1.44, 1.39, 1.48, 1.82, and 0.63 Å, respectively. However, C3larvinA has an extended N-terminus that protrudes beyond helix 1 and is longer than the N-terminus of both C3bot1 and C3bot2. A conserved residue, Phe^9^ (C3bot1 numbering; Phe^23^ in C3larvinA) in this extended N-terminus is found in nearly all C3-like toxins except for C3larvin_trunc_ and C3cer. A second residue, Thr^10^ (C3bot1 numbering; Glu^25^ in C3larvinA) is conserved among all C3-like toxins except those from *P. larvae* (Figure 1A). Thr^10^ is substituted with Ile in Plx2A and a Glu residue in C3larvinA. Multiple sequence alignment between the N-termini of both C2-like and C3-like toxins show distinct clusters of conserved residues (Figure 1D). These C3/C2-like toxins consist of enzymes that have C3-like activity, but for which a B-domain has been found. Despite their differences, there are highly conserved residues among all groups. This is interesting, since the N-terminal adaptor domain of the iota I_a_-subunit (PDB: 4GY2) is required to bind to the translocating I_b_-domain (B-domain) via its N-terminal Ca^2+^-binding motif [54,55]. This may point to a divergent evolution of C3-like toxins from an ancestral C2-like toxin where the binding machinery evolved to accommodate a single-domain enzyme [56].

Overall, the active site of C3larvinA is remarkably like C3bot1, with the location of the β-strands appearing nearly identical between the two structures. An interesting feature in C3larvinA is the presence of extended β-strands (β-5, β-6, β-7), the most significant being the β-sheet (β-5) on the N-terminal region of the ARTT-loop and the β-sheet (β-6) containing the Q-X-E motif (data not shown). As a result, the ARTT-loop has comparatively fewer residues than most C3-like toxins and may provide more stability within the active site. Also, the solvent-exposed loops, i.e., the ARTT- and PN-loops in C3larvinA, adopt a more open conformation compared with other C3-like structures (Figure 2A). The catalytic Glu^189^ is in a similar position to that of other C3-like toxins. However, the catalytic Gln^187^ of C3larvinA like C3larvin_trunc_ (PDB: 4TR5) has a significantly different orientation than the conserved Gln residues found in other NAD^+^-bound conformations of C3-like toxins (Figure 2 B). The Gln^187^ in its current position would clash with the residues of the PN loop of other C3-like toxins. It is rotated along the axis of the main chain, moving ∼4 Å farther than the similar residue (Gln^172^) in C3bot1. The source of this change in orientation may lie in the residue separating the Gln and Glu (Q-X-E motif). In other solved structures, the residue which separates these catalytic residues is a Leu (or another Gln, in the case of C3staus 1, 2, and 3). C3larvinA is like C3larvin_trunc_ in which the Q-X-E intervening residue has been substituted with a Tyr (Tyr is also present in this position in C3cer); the intervening residue in Plx2A is a Leu. This Tyr residue found in C3larvinA may exert some steric hindrance with residues in nearby areas of the protein and cause a displacement of the backbone structure in this area. It is also possible that there is an induced-fit mechanism in this region of the structure, in which binding of a substrate would cause the Glu^189^ to shift to a more catalytically relevant position. Upon binding an NAD^+^ molecule, the ARTT-loop changes its conformation from a solvent-exposed environment to a more buried conformation in C3bot1. In particular, the Gln^172^ residue in C3bot1 makes a large shift (∼8 Å) toward the interior of the NAD^+^-binding cleft. Interestingly, the position of Gln^189^ residue in C3larvinA is also in a similar location and orientation (Figure 2B).

### C3larvinA–NADH–RhoA model

**Figure 3:**
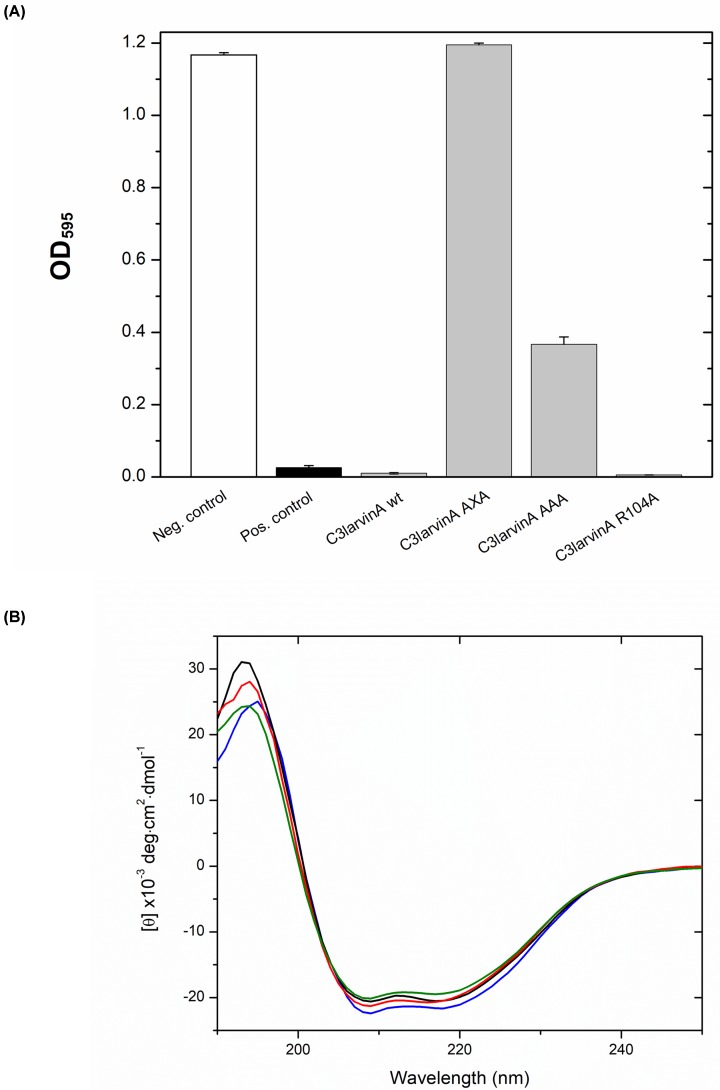
C3larvinA inhibition of yeast growth and CD spectra (**A**) Inhibition of yeast growth by C3larvinA and selected catalytic variants. All growth is compared with that of yeast expressing a positive control toxin, *P. aeruginosa* exotoxin A catalytic domain. The negative control contained the empty plasmid, pRS415 CUP1. The C3larvinA variants are shown as C3larvinA AXA (Q187A-X-E189A), C3larvinA AAA (S149A-T150A-S151A) and C3larvinA R104A. (**B**) CD spectra of C3larvinA WT (red), Q187A-X-E189A (green), S149A-T150A-S151A (black) and R105A (blue) were collected in aqueous solution (25°C) containing 250 mM NaF and 10 mM Tris, pH 7.5 buffer. The concentration of the proteins was at 0.16 mg/ml and each protein CD consensus spectrum was obtained by scanning from 250 to 190 nm and is the average of nine independent spectra.

A homology model of C3larvinA–NADH–RhoA was built based on the template of the C3cer-NADH–RhoA crystal structure (PDB: 4XSG) using Phyre2 based on hidden Markov models and detection by HHpred 1.51 [41] (Figure 2C). The surface contact area between C3larvinA and RhoA is large (∼1200 Å^2^) and the toxin recognizes RhoA via the switch I, switch II, and interswitch regions as seen in the C3cer–RhoA crystal structure. C3larvinA is highly conserved (nearly identical) with C3cer in the loop regions that make contact with RhoA (Figure 2D); these loop regions include L2 (residues 45–52, active-site loop), L3 (residues 100–110, adenine loop); L4 (residues 148–156, PN-loop), L5 (residues 175–183, ARTT-loop), L6 (residues 204–209). As observed for the C3cer–RhoA complex structure, there is no L1 (Tyr^60^–Tyr^62^) in C3larvinA as seen in the iota I_a_ complex with actin (PDB: 3BUZ) [57]. In the C3cer–RhoA structure, Tyr^180^ in turn 1 of the ARTT-loop recognizes RhoA via a hydrophobic patch around the ADP-ribose acceptor residue, Asn^41^ in RhoA. This residue is conserved in C3larvinA (Tyr^184^) indicating that it interacts and catalyzes the modification of Asn^41^ in RhoA in an identical manner to C3cer (Figure 2D). Gln^183^ in turn 2 (Gln-X-Glu motif) also interacts with Asn^41^ in RhoA in the C3cer complex and the same residue and motif is found in C3larvinA (Gln^187^-X-Glu^189^). Interestingly, the intervening ‘X’ residue in the Gln-X-Glu motif in both C3cer and C3larvin is a Tyr residue further cementing their identical catalytic signatures and detailed mechanism for RhoA modification. As observed for C3cer, the Tyr^184^ in C3larvinA interacts with a patch on RhoA composed of Val^43^, Ala^56^, and Trp^58^. The hydroxyl group of Tyr^184^ forms an H-bond with the main-chain carbonyl group of Leu^57^ in RhoA. RhoA Asn^41^ forms an H-bond with Gln^187^ in the Q-X-E motif with the ARTT-loop of C3larvinA. Clearly, Gln^187^ and Glu^189^ participate in the ADP-ribose transferase reaction to RhoA Asn^41^ based on the mutagenesis of this motif in C3larvinA, which resulted in a loss of enzyme function that restored the growth-deficient phenotype in yeast (variant C3larvinA AXA in Figure 3A). Additionally, Asp^175^ in C3cer interacts with the critical Arg^5^ in RhoA at 2.7 Å and this residue is also conserved in C3larvinA (Asp^179^).

### C3larvinA shows strong yeast cytotoxicity

To test the toxicity of C3larvinA to eukaryotic cells, a yeast-based growth-inhibition assay was employed [48]. In this method, C3larvinA gene expression is under control of the CUP1 (copper-inducible) promoter, and an active toxin will cause a growth-defect phenotype in yeast as previously shown for two other C3-like toxins from *P. larvae* [37,38]. The effect of C3larvinA WT and active-site variants on yeast growth is shown in Figure 3A. This test showed that a weak C3larvinA WT gene-induction by copper in the yeast culture (0.5 mM) was highly toxic to yeast cells, even more so than ExoA (catalytic domain) from *P. aeruginosa* (positive control toxin) (Figure 3A). The R104A variant did not recover the growth-defect phenotype when expressed in yeast, whereas the STS motif variant (S149A/T150A/S151A) partially restored the growth defect (Figure 3A). Notably, the C3larvinA Q-X-E catalytic motif variant (Q187A-X-E189A) fully restored the C3larvinA growth defect in yeast. This pattern was also observed for the Q-X-E variant for C3larvin_trunc_ [38]; however, the STS and NAD^+^-binding Arg motifs were not tested in this assay for C3larvin_trunc_. In the case of Plx2A, a major virulence factor of *P. larvae* ERIC I genotype that causes contemporary AFB outbreaks worldwide, the pattern of growth restoration in the catalytic signature is different from C3larvinA [37]. First, the Q-X-E catalytic motif variant was similar for both the Plx2A and C3larvinA toxins. However, the STS motif and NAD^+^-binding Arg motif Plx2A variants both completely recovered the growth defect in yeast [37]. The origin of these differences in the catalytic signature variants is not known, but may be related to the extended N-terminus in C3larvinA compared with Plx2A. C3cer toxin was previously shown to lose all transferase activity when either the catalytic Gln or Glu were substituted with an Ala residue [53]. This suggests that the three *P. larvae* C3-like toxins possess a highly specialized catalytic site, where both the Gln and Glu residues in the Q-X-E catalytic motif are essential for proper function. Thus, these results indicate that C3larvinA is a *bona fide* mART toxin and confirmed its cytotoxicity (caused by its mART activity) in a model (yeast) eukaryotic system.

The C3larvinA enzyme is a more stable protein in aqueous solution with a *T*_m_ value of 63°C (Table 1) compared with 55°C for Plx2A [37] and 51°C for C3larvin_trunc_ (unpublished data). These thermal stability data indicate that a full-length helix 1 (Plx2A; Figure 1A) adds folded stability to the enzyme compared with the truncated helix 1 in C3larvin_trunc_ and the ADPRT fold is further stabilized in C3larvinA which harbors an additional 19 residues extending past helix 1 compared with Plx2A (Figure 1A).

### C3larvinA binds and hydrolyzes NAD^+^ as substrate

C3larvinA has only two Trp residues (Trps 33 and 41; Figure 1A) and both are located within Helix 1 with Trp^33^ being a conserved Trp found in both C2- and C3-like toxins (Figure 1D). Trp^41^ in C3larvinA is conserved in the *P. larvae* C3-like toxins (Figure 1D). Both Trp residues face inwardly in the protein folded structure and likely provide stability to the ADPRT fold through numerous molecular packing interactions (data not shown). The Trp fluorescence was exploited to characterize the NAD^+^ substrate binding to the active site for C3larvinA WT and variants (Table 2). In these experiments, C3larvinA proteins were titrated with NAD^+^ substrate which caused quenching of the intrinsic Trp fluorescence (data not shown) and binding isotherms (single-site model) were used to calculate the *K*_D_ values for NAD^+^ of approximately 56 µM for the WT protein (Table 2). This affinity for the NAD^+^ substrate compares well with the affinity shown by other C3-like toxins such as C3bot1, Plx2A and C3larvin_trunc_ of 60, 33, and 21 µM, respectively [37,38,58].

**Table 2:** Binding affinity and GH turnover number for C3larvinA and catalytic variants against NAD^+^

Protein	*K*_D_ (µM)^1^	*k*_cat_ (min^−1^)^1^
C3larvinA	56 ± 11	261 (±20) × 10^−3^
C3larvinA AAA	34 ± 3	9.5 (±2) × 10^−3^
C3larvinA R105A	63 ± 13	5.3 (±0.1) × 10^−3^
C3larvinA AXA	143 ± 13	7.7 (±0.2) × 10^−3^

1 The measurements of the kinetic and substrate-binding affinity parameters for C3larvinA are described in the ‘*Experimental procedures*’ section. The parameters for NAD^+^ substrate binding and GH enzyme activity represent the mean ± SD of at least three different measurements.

### C3larvinA GH activity

GH activity is present as a secondary enzymatic activity in most mART enzymes, is likely not biologically relevant and represents an alternative reaction where OH^−^ serves as the nucleophile in the absence of a target protein [59]. C3larvinA GH activity was characterized with a fluorescence-based assay developed previously (Table 3) [60]. C3larvinA GH activity showed Michaelis–Menten behavior and gave a *K*_M_ value of 107 ± 20 μM and a *k*_cat_ of 261 ± 20 × 10^−3^ min^−1^ (Table 3). C3 enzymes show a range of kinetic parameters for GH activity; for example, C3lim had a *K*_M_ (NAD^+^) = 160 μM and a *k*_cat_ = 2 × 10^−3^ min^−1^ [61]. C3-like toxins from *P. larvae*, C3larvin_trunc_ and Plx2A, had *K*_M_ values of 120 and 176 μM, respectively and *k*_cat_ values of 1.3 × 10^−3^ and 58 × 10^−3^ min^−1^, respectively [37,38].

**Table 3:** Kinetic parameters for GH and transferase activity of C3larvinA

Parameter^1^	GH	Transferase	
		NAD^+^	RhoA-GST
*K*_M_ (µM)	107 ± 20	75 ± 10	5.3 ± 1.0
*k*_cat_ (min^−1^)	261 (±20) × 10^−3^	61 ± 3	
*k*_cat_/*K*_M_ (M^−1^.min^−1^)	2.44 × 10^3^	8.13 × 10^5^	1.15 × 10^7^

Kinetic parameters for transferase activity are shown for the RhoA target substrate.

1 The measurements of the kinetic parameters for C3larvinA GH and transferase activity are described in the ‘*Experimental procedures*’ section. The parameters represent the mean ± SD of at least three different measurements.

### C3larvinA active-site variants

The active-site of C3larvinA was probed for the presence of C3-like toxin hallmark catalytic motifs via site-directed mutagenesis as reported for both C3larvin_trunc_ [38] and Plx2A [37]. The STS variant (S149A/T150A/S151A) showed a slightly higher affinity for NAD^+^ (34 µM), the R105A variant had a similar affinity (63 µM) and the A-X-A variant (Q187A-X-E189A) showed a weaker affinity (143 µM) for the NAD^+^ substrate than the WT enzyme (Table 2). Notably, this change in NAD^+^ affinity pattern was not seen in the comparable Plx2A variants [37] and likely reflects specific differences in the stability of the C3larvinA active site compared with Plx2A. The GH activity of all three C3larvinA catalytic variants, S149A/T150A/S151A, Q187A-X-E189A, and R105A was weak compared with the WT enzyme (5.3–9.5 × 10^−3^ min^−1^ compared with 261 × 10^−3^ min^−1^; Table 2). To assess folded integrity of the C3larvinA variants, CD spectroscopy was conducted on the WT C3larvinA and compared with the spectra of the variants, S149A/T150A/S151A, Q187A-X-E189A, and R105A. The WT enzyme showed a typical CD spectrum for an α/β-type domain characteristic of the ADPRT fold in mART toxins (Figure 3B). Importantly, there were no significant differences in the folded integrity of the C3larvinA WT and three catalytic variants based on their CD spectra (Figure 3B). This indicates that C3larvinA was properly folded and was an active enzyme and that site-directed mutagenesis of the catalytic signature produced variants that were also properly folded (Figure 3B, Tables 2 and 3).

### C3larvinA ADP-ribosylates RhoA

All C3-like toxins have been shown to target RhoA as the primary substrate and have also been shown to target both RhoB and RhoC. Notably, the *Staphylococcus aureus* C3-like toxins, C3stau1, 2, and 3, have also been shown to have several other secondary substrates [62,63]. Previously, it was determined that human RhoA is a good substrate for *P. larvae* C3-like toxins, C3larvin_trunc_ and Plx2A, which serves as a substrate homolog for Rho1 in honey bee larvae [37,38]. Previously, a constitutively active RhoA-GST variant, with a deleted CAAX motif, was shown to be an active GTPase and was used as the substrate protein for C3larvin_trunc_ and Plx2A [37,38].

RhoA was confirmed as the target for C3larvinA transferase activity by using fluorescein-NAD^+^ as an NAD^+^ substrate analog. The addition of the fluorescein group to the adenine ring of the NAD^+^ molecule allows for visualization of the ADP-ribose moiety covalently attached to a protein resolved on an SDS/PAGE gel; in this case, it revealed that RhoA is also a substrate for C3larvinA (Figure 1C, lanes 9 and 10). An HPLC-based assay was devised to measure the transferase activity of C3larvinA against RhoA-GST as the target substrate with NAD^+^ as the donor substrate. Based on the HPLC assay results, C3larvinA was found to have *K*_M_ values of 75 and 5.3 µM for the NAD^+^ and RhoA-GST substrates, respectively, with a *k*_cat_ value of 61 min^−1^ for transferase activity, which is ∼230-fold higher than its GH activity (Tables 2 and 3). The enzyme specificity constant (k_cat_/K_M_) is a useful measure to compare a family of related enzymes and their specificity toward a given substrate or series of substrates. The higher the value of the specificity constant, the more specific is an enzyme toward a common substrate. The specificity constant for C3larvinA transferase activity toward the RhoA-GST ADP-ribose acceptor substrate is shown in Table 3. C3larvinA gave a value of 1.15 × 10^7^ M^−1^.min^−1^ for RhoA-GST, which is a modest value for an enzyme–substrate reaction. However, RhoA-GST is a rather large substrate (∼48 kDa) compared with many small-molecule substrates for common enzymes and its diffusion is rather slow and will undoubtedly contribute to the smaller specificity constant (second-order rate constant) for this enzyme–substrate pair. Importantly, C3larvin_trunc_ and Plx2A are not as efficient as C3larvinA in their transferase function involving RhoA-GST as the acceptor substrate with specificity constants of 3.13 × 10^5^ and 7.19 × 10^6^ M^−1^.min^−1^, respectively.

### C3larvinA interferes with actin remodeling in macrophages

**Figure 4:**
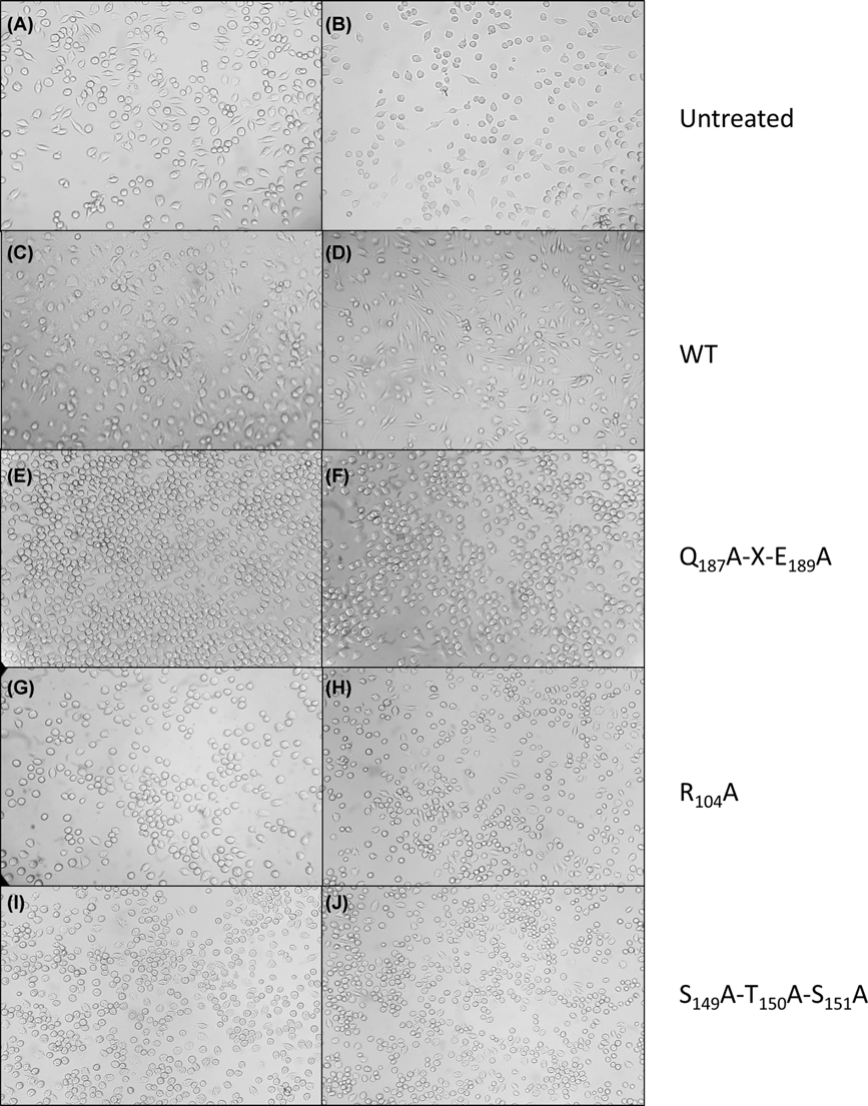
The effect of C3larvinA on cultured macrophages Cell morphology assays were performed with J774A.1 mouse macrophage cells that were grown to confluence in 25-cm^2^ culture flasks, the cells resuspended, and 100 μl was transferred to 6- or 96-well culture plates containing 4 ml supplemented medium (200 μl in the case of the 96-well plates). The cells were left for 48 h to grow in the new medium, at which point either toxin or control buffer was added. The cells were observed 20 h later and any morphology changes were recorded. (**A**) Untreated macrophage cells, (**B**) cells with buffer only; cells treated with: (**C**) 30 nM WT C3larvinA, (**D**) 300 nM WT C3larvinA, (**E**) 30 nM C3larvin A Q187A-X-E189A, (**F**) 300 nM C3larvinA Q187A-X-E189A, (**G**) 30 nM C3larvinA R104A, (**H**) 300 nM C3larvinA R104A, (**I**) 30 nM C3larvinA S149A-T150A-S151A and (**J**) 300 nM C3larvinA S149A-T150A-S151A. The elongated protrusions from the cells visible in (C) and (D) are phenotypic changes indicative of infection with C3 toxins. These protrusions are visibly absent for C3larvinA variant-treated cells (E–J).

C3larvinA cell entry was tested against the J774A.1 macrophage cell line derived from mice. C3bot1 and C3lim can enter these cells at nanomolar concentrations [64]. Previous work suggested that the N-terminal helices of C3bot1 and C3lim may be important for their cell entry [64]. Both toxins were previously shown to cause distinct morphology changes in macrophages as seen by enlarged cells with filopodia-like protrusions, with the most obvious changes occurring in J774A.1 cells [64]. We previously showed that Plx2A caused cell morphological changes in murine macrophages indicative of interference with actin cytoskeletal processes [37]. Under identical conditions, WT C3larvinA caused macrophages to elongate in a toxin-dependent manner, indicative of toxin commandeering of RhoA-dependent cytoskeletal functions, such as actin remodeling (Figure 4). The three catalytic variants of C3larvinA, S149A/T150A/S151A, Q187A-X-E189A, and R105A, showed marginal effects on macrophage morphology, as expected. This demonstrates that C3larvinA causes actin remodeling in the target macrophage cells via its ADP-ribosyltransferase activity against RhoA.

### C3larvinA interferes with cytokinesis in insect cells

**Figure 5:**
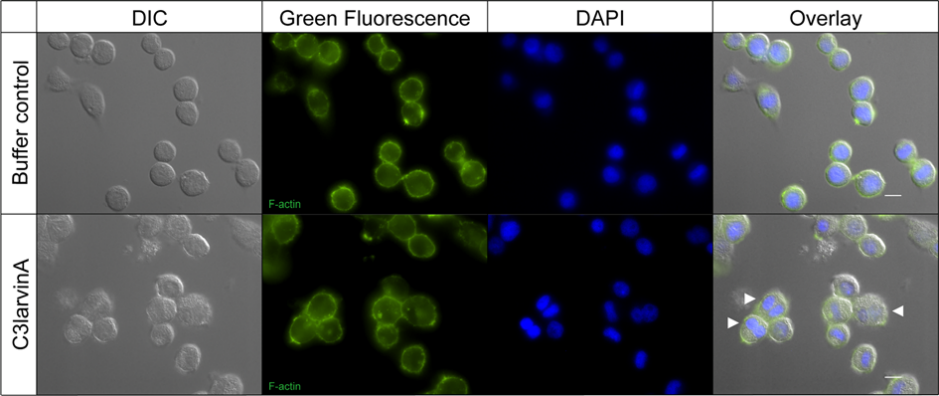
Effect of C3larvinA from *P. larvae* 11-8051 on insect cells Lepidopteran Sf9 cells were incubated in the presence of C3larvinA from *P. larvae* 11-8051 (final concentration near 1.0 µM). Staining of the cytoskeleton with FITC-labeled phalloidin revealed no effect on cytoskeleton organization. Bi-nucleated cells indicating an interference in cytokinesis become visible after DAPI staining (scale bars: 10 µm).

We also tested the effect of purified C3larvinA on the insect cell line Sf9 from the fall armyworm, *Spodoptera frugiperda* (Lepidoptera). We chose this model system because to the best of our knowledge, there is no viable honey bee cell line currently available. In this assay, an effect of C3larvinA (final concentration approximately 1.0 µM) on Sf9 cells was observed similar to the effect of Plx2A on the same cell line [37]. Sf9 cells treated with purified C3larvinA exhibited a phenotype with slightly enlarged, binucleated cells indicating an interference in cytokinesis (Figure 5, white arrows). Like Plx2A, C3larvinA did not cause an effect on the actin cytoskeleton of insect cells as visualized with phalloidin staining (Figure 5). These results point to a remarkable difference in RhoA signaling in mammalian versus insect cells.

## Discussion

C3larvinA and B were recently identified as a binary toxin pair that function as a virulence factor in *P. larvae* ERIC III/IV strain 11-8051 [39], which was originally isolated from Chilean honey [65]. In the present study, C3larvinA (29 kDa) was shown to be a typical C3-like mART toxin with the classical ADPRT-fold and catalytic signatures. Recombinant C3larvinA was expressed and purified from *E. coli* and was shown to possess both GH (OH^−^ from water as the nucleophile) and transferase activities (Asn^41^ from RhoA as the nucleophile) (Figure 1C, Tables 2 and 3). C3larvinA is similar to previously characterized C3-like mART toxins and is most similar to the *P. larvae* C3-like toxins, all of which modify RhoA at Asn^41^ [37,38,52,53]. The characterization of the GH activity using a fluorescent NAD^+^ analog revealed that C3larvinA follows Michaelis–Menten kinetics with respect to the NAD^+^ substrate. Kinetic parameters were determined for the GH activity, and were similar to those previously determined for C3-like toxins [37,38,52]. Transferase kinetic parameters to the RhoA substrate were also determined quantitatively using a novel HPLC-based method. Site-directed mutants of the active-site architecture of C3larvinA confirmed its structure and function as a C3-like mART enzyme, including a Q-X-E motif involved in the transferase reaction of ADP-ribose to Asn^41^ in RhoA, a catalytic Arg residue involved in docking/orientation of the NAD^+^ substrate and an STS motif required for stabilization of the NAD^+^-binding pocket [66]. Amino-acid residue substitution of each of these catalytic signature motifs caused a near total loss of C3larvinA GH activity (Table 2).

The expression of WT C3larvinA in the cytoplasm of yeast demonstrated that it is cytotoxic to a eukaryotic host as previously shown for both C3larvin_trunc_ and Plx2A toxins [37,38]. Notably, the catalytic variant (Q187A-X-E189A) was not cytotoxic as expected; furthermore, the NAD^+^-binding variants of C3larvinA showed a surprising level of cytotoxicity toward yeast, with the S149A/T150A/S151A variant only showing a 30% reduction in cytotoxicity compared with the WT. Remarkably, the R105A variant was as cytotoxic as the WT toxin in yeast (Figure 3A) which does not correlate with the GH activity data (Table 2). The basis for this unexpected finding is currently not known and will require further investigation.

Previously, we determined the crystal structures of two *P. larvae* C3-like toxins, an N-terminal truncated C3larvin_trunc_ (PDB:4TR5) [38] and Plx2A, an important virulence factor in *P. larvae* ERIC I strains (PDB: 5URP) [37]. C3larvinA from *P. larvae* ERIC III/IV strain 11-8051 shares 55% sequence identity with Plx2A (Figure 2B). Consequently, we built a homology model of C3larvinA with NAD^+^ substrate based on the Plx2A structure (PDB: 5URP), which clearly showed that C3larvinA has an extended N-terminus compared with Plx2A (Figure 2A). The role of the N-terminal helix 1 in these *P. larvae* toxins was further supported by macrophage cell entry experiments. Previously, the truncated C3larvin_trunc_ toxin was unable to enter macrophages because it lacks part of helix 1 (Figure 1A,D), whereas a C3larvin–C3bot1 chimera possessing the C3bot1 N-terminal sequence (Tyr^2^–Trp^18^) was capable of entering macrophages [38]. In contrast, Plx2A from *P. larvae* was fully functional and entered macrophages causing the expected cell enlargement and elongation with filipodia-like extensions [37]. C3larvinA with its elongated N-terminus entered macrophages and gave the expected C3-like phenotype (Figure 4) at a similar dose as seen for Plx2A. This suggests that the extended N-terminus in C3larvinA may play an additional role in its cell function since it does not modulate cell entry compared with the shorter Plx2A toxin. In this context, C3larvinA with its extended N-terminus is considerably more stable than either C3larvin_trunc_ or Plx2A, but the role of the N-terminal extension is currently not known.

A homology model of C3larvinA in complex with RhoA (Figure 2C) built on the C3cer-RhoA crystal structure [40] suggested that C3larvinA shares identical catalytic features for ADP-ribose transfer to RhoA and a highly similar catalytic mechanism. Furthermore, sequence alignment of C3larvinA with C3cer (Figure 2D) showed that their catalytic signatures, including the contact loop regions (L2–L6) are highly conserved (∼50% identity; 98% similarity). The key catalytic residues involved with ADP-ribose transfer to Asn^41^ in RhoA are identical between C3cer and C3larvinA indicating that these two C3-like toxins share an identical catalytic mechanism in the C3-like toxin subgroup.

Furthermore, C3larvinA was able to enter lepidopteran Sf9 cells as was previously shown for Plx2A [37]. The addition of purified C3larvinA to the Sf9 cells caused an enlargement of the cells and the presence of two nuclei indicative of perturbation of cytokinesis as previously observed for Plx2A [37]. It is known that members of the Rho subfamily of low molecular weight GTP-binding proteins participate in cell cycle progression. In particular, switching of RhoA between the activated and inactivated state is required for completion of cytokinesis [67–69]. In contrast with murine macrophage cells, C3larvinA did not influence the actin cytoskeleton as was also shown for Plx2A [37]. The ADP-ribosyltransferase toxins from *P. larvae*, C3larvinA and Plx2A, both were able to enter non-phagocytic insect cells pointing to a difference in cell entry to the other C3-like toxins, which have mammalian cells as their natural host. Interestingly, C3larvinA and Plx2A are the only functional C3-like toxins with a B-subunit [39,50]. This deserves further investigation. The kinetic data and cell culture assays in this study show that C3larvinA is more active than C3larvin_trunc_ and show that C3larvinA can enter cells on its own in contrast with C3larvin_trunc_. This substantiates the hypothesis that only C3larvinA from *P. larvae* ERIC III/IV strain, 11-8051, is the active form of this toxin. Furthermore, C3larvinA has an intact B-subunit partner which also has been shown to play a role in virulence in *P. larvae* 11-8051 [39]. Notably, the interaction between A- and B-subunits is an interesting subject for future studies.

## Abbreviations

ADPRT: ADP-ribosyltransferase
AFB: American Foulbrood
CD: circular dichroism
ERIC: enterobacterial-repetitive-intergenic-consensus
GB/VI: generalized born-volume integral
GH: glycohydrolase
HPLC: high-performance liquid chromatography
IMAC: immobilized-metal-affinity chromatography
IPTG: isopropyl β-D-1-thiogalactopyranoside
mART: mono-ADP-ribosyltransferase
MD: molecular dynamics
MOE: Molecular Operative Environment
NAMD: Nanoscale Molecular Dynamics
PABA: para-4-nitrobenzoic acid
PDB: Protein Data Bank
Phyre2: **P**rotein **H**omology/**A**nalog/**Y R**ecognition **E**ngine
RMS: root-mean-square
SD: standard deviation
SD-leu: single dropout formulation (without leucine) of Synthetic Defined Media
SEC: size-exclusion chromatography
vdW: van der Waals
WT: wild-type
ε-NAD^+^: etheno-NAD^+^
